# Seeing beyond words: nanotechnology in hepatocellular carcinoma - a bibliometric study

**DOI:** 10.3389/fonc.2024.1487198

**Published:** 2025-01-15

**Authors:** Talaiti Tuergan, Aimitaji Abulaiti, Alimu Tulahong, Ruiqing Zhang, Zhongdian Yuan, Yanze Lin, Yingmei Shao, Tuerganaili Aji

**Affiliations:** ^1^ Hepatobiliary and Echinococcosis Surgery Department, Digestive and Vascular Surgery Center, First Affiliated Hospital of Xinjiang Medical University, Urumqi, China; ^2^ State Key Laboratory of Pathogenesis, Prevention and Management of High Incidence Diseases in Central Asia, Xinjiang Medical University, Urumqi, China; ^3^ Xinjiang Clinical Research Center for Echinococcosis and Hepatobiliary Diseases, First Affiliated Hospital of Xinjiang Medical University, Urumqi, China

**Keywords:** nanotechnology, hepatocellular carcinoma, bibliometrics, drug delivery, apoptosis

## Abstract

**Background:**

Nanotechnology has increasingly been applied in the diagnosis and treatment of hepatocellular carcinoma (HCC) over the past two decades. This study aims to explore the utilization of nanotechnology in HCC through a bibliometric analysis, identifying key themes, trends, and contributions in this field.

**Methods:**

The study utilized VOSviewer and CiteSpace software to perform a bibliometric analysis, evaluating scholarly contributions related to nanotechnology in HCC. The analysis focused on co-occurrence network relationships, publications, citations, and contributions from various entities and authors.

**Results:**

The analysis revealed a total of 2,968 articles, with China and the USA being the most prominent contributors in terms of publications and citations. Notable contributions were made by the Chinese Academy of Sciences and authors Gao Jie and Li Yan. LLOVET JM emerged as the most co-cited author, indicating a leadership role in the field. The “International Journal of Nanomedicine” was identified as the leading publisher, while “Biomaterials” ranked highest in citations. The research mainly focused on drug delivery systems and apoptosis, highlighting significant advancements in utilizing nanotechnology for HCC treatment.

**Conclusion:**

This bibliometric study underscores the critical role of nanotechnology in advancing the diagnosis and treatment of hepatocellular carcinoma, with a particular emphasis on drug delivery and apoptosis. The findings highlight the contributions of key countries, institutions, and authors, reflecting the global effort and collaboration in this rapidly evolving field.

## Introduction

Hepatocellular carcinoma (HCC) poses a significant global public health burden, being identified by the World Health Organization as the second most common cause of cancer-related mortality worldwide, with an annual toll exceeding 800,000 deaths (1, 2). The prevalence of this malignancy is notably pronounced in regions such as Asia and sub-Saharan Africa, where it is closely linked to the elevated incidence of hepatitis virus infections (3). The management of HCC is multifaceted, encompassing conventional modalities such as surgery, radiation therapy, and chemotherapy (4). Nevertheless, a considerable number of patients present with advanced disease, thereby compromising the efficacy of these therapeutic interventions (5). Furthermore, the frequent recurrence of HCC and the substantial burden of adverse effects associated with treatment substantially impact the quality of life of affected individuals (6). In this particular context, nanotechnology demonstrates significant promise as a result of its ability to enhance drug bioavailability, selectively target tumor tissues, regulate drug release, employ synergistic treatment approaches, enable early diagnosis and real-time monitoring, mitigate multidrug resistance, and bolster immune response (7). These attributes position nanotechnology as a hopeful prospect for the future management of HCC (7, 8).

The current research in nanotechnology for the treatment of HCC is progressing rapidly and showcasing a multitude of innovative applications (7). Nanoparticles, when integrated into drug delivery systems, have the capability to accurately transport chemotherapy drugs, gene therapy agents, and other therapeutic molecules to liver cancer cells, ultimately enhancing the precision and effectiveness of treatment (9). For instance, nanoparticles exhibit tailored responses to the tumor microenvironment, including sensitivity to pH and temperature, thereby facilitating targeted drug delivery and minimizing harm to healthy cells (10). Furthermore, nanotechnology holds promise for the early detection and surveillance of HCC. Tailored nano-probes can be engineered to identify and adhere to liver cancer-specific biomarkers, facilitating early lesion detection and continuous treatment monitoring through advanced imaging modalities (11). The advancement of these technologies not only enhances the promptness and efficiency of therapeutic interventions but also holds promise for the customization of HCC management strategies in forthcoming clinical practice (7, 9–13). The advancement of these technologies, coupled with the ongoing progress of clinical trials, is anticipated to result in significant advancements in the treatment of HCC through the application of nanotechnology in the foreseeable future (14).

The expanding utilization of nanotechnology in the diagnosis and treatment of HCC has led to a corresponding growth in the research literature (15). Bibliometrics, a quantitative method for analyzing scientific literature, can efficiently assess and consolidate a vast array of research findings, elucidating trends and patterns in the field of study (16, 17). This study aims to conduct a systematic review and analysis of the research literature on nanotechnology in the context of HCC diagnosis and treatment, utilizing bibliometric methods to identify key research areas, trends, potential gaps, and future research directions. This paper will investigate the evolution of nanotechnology in the diagnosis and treatment of HCC by examining pertinent literature from databases. It will focus on the development trajectory, prominent research institutions, leading scientists, and current research trends and advancements in this field.

This paper seeks to offer a scholarly contribution by providing a reference point and guidance for future research endeavors, thereby fostering the advancement of nanotechnology in the realm of HCC treatment. Furthermore, it serves to enlighten policymakers and research funding agencies on the evolving landscape of research within this domain, facilitating more efficient allocation of resources and formulation of research policies. In conclusion, the growing significance of nanotechnology in the treatment of HCC is underscored by its ongoing progress and broadening scope of application (18). This paper employs a bibliometric approach to systematically examine the research trends and advancements in nanotechnology for the treatment of HCC, offering scientific analysis and valuable insights for future research and practical applications.

## Materials and methods

### Data source

**Figure 1:**
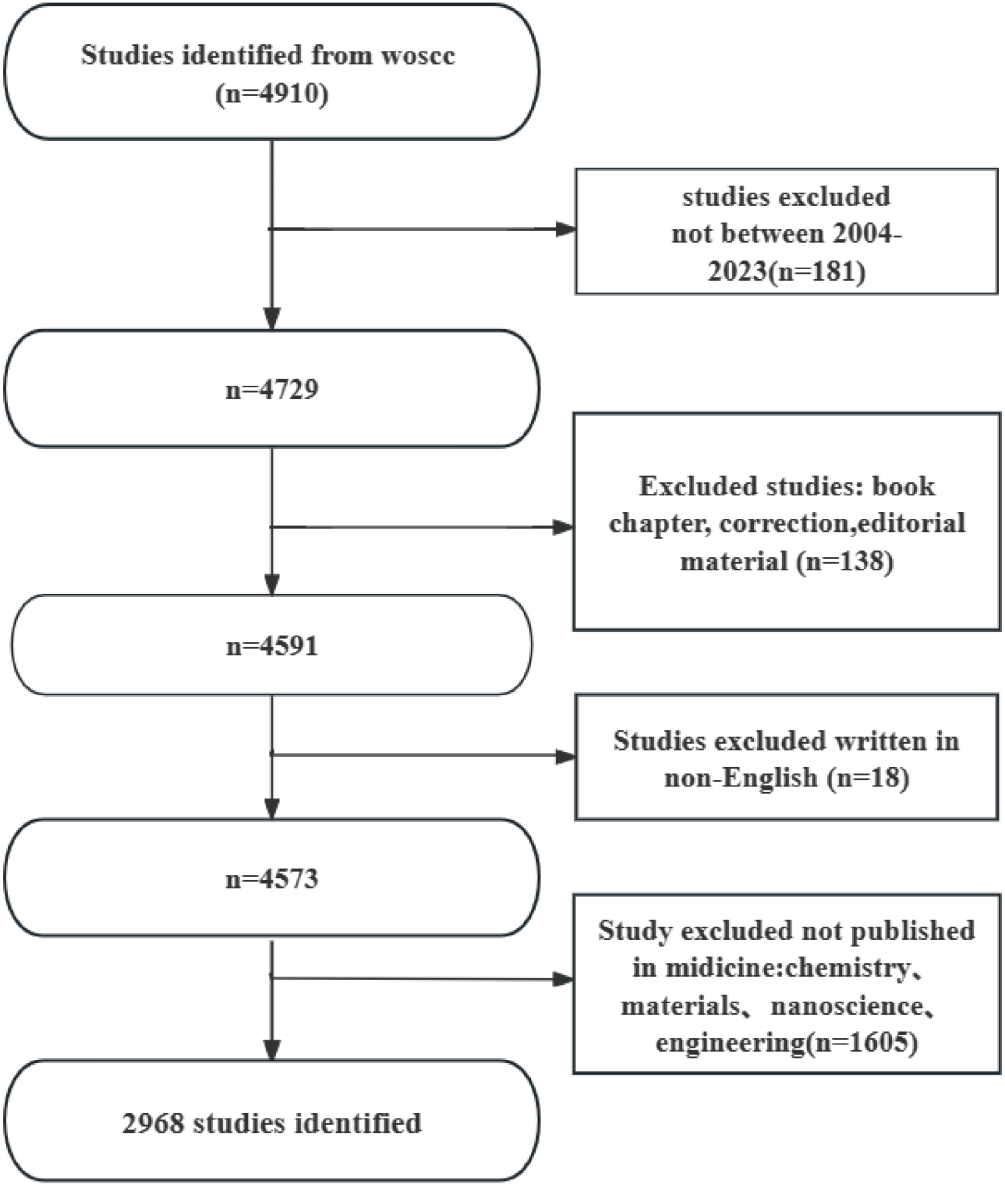
Flowchart of this study.

Given the superior accuracy of document type identification in the Web of Science Core Collection (WoSCC) database compared to other databases, and its widespread recognition as the preferred resource for literature analysis, we opted to conduct our retrieval work within this database (19, 20). We conducted our search within the Science Citation Index Expanded (SCIE) sub-database of the Web of Science Core Collection, as this database comprehensively covers high-quality publications in the fields of natural sciences and medicine, which align with our research focus on nanotechnology applications in HCC. On April 12, 2024, we conducted a search for all articles pertaining to the utilization of nanotechnology in the treatment of HCC within the WoSCC database, spanning the time period from January 1, 2004, to December 31, 2023. The search formula utilized is as follows: TS = (nanodot* OR nanoparticle* OR nanomaterial* OR nanotube* OR nanosheet* OR “quantum dot*” OR nanofiber* OR nanosphere* OR nanorod* OR nanowire* OR nanocrystal* OR nanocomposite* OR nanodevice* OR nanocluster* OR nanotechn* OR nanocarrier* OR nanowire* OR nanoliposome* OR nanoemulsion* OR nanocrystal* OR nanoconjugate* OR nanogels* OR nanodiamond* OR nanoporou* OR nanosilver* OR nanopore* OR nanomicell* OR nano size* OR nanomedicine* OR nanofibrou*) AND ((((((((((((((((((TS=(“Carcinoma, Hepatocellular”)) OR TS=(“Carcinomas, Hepatocellular”)) OR TS=(“Hepatocellular Carcinomas”)) OR TS=(“Liver Cell Carcinoma, Adult”)) OR TS=(“Liver Cancer, Adult”)) OR TS=(“Adult Liver Cancer”)) OR TS=(“Adult Liver Cancers”)) OR TS=(“Cancer, Adult Liver”)) OR TS=(“Cancers, Adult Liver”)) OR TS=(“Liver Cancers, Adult”)) OR TS=(“Liver Cell Carcinoma”)) OR TS=(“Carcinoma, Liver Cell”)) OR TS=(“Carcinomas, Liver Cell”)) OR TS=(“Cell Carcinoma, Liver”)) OR TS=(“Cell Carcinomas, Liver”)) OR TS=(“Liver Cell Carcinomas”)) OR TS=(“Hepatocellular Carcinoma”)) OR TS=(“Hepatoma”)) OR TS=(“Hepatomas”)。The literature review methodology employed in this study adheres to specific inclusion and exclusion criteria. Inclusion criteria encompass full-text publications in English pertaining to the utilization of nanotechnology in the context of HCC, published between January 1, 2004, and December 31, 2023. Exclusion criteria encompass topics not directly related to the application of nanotechnology in HCC, articles unrelated to the field of medicine, and non-peer-reviewed sources such as conference abstracts, news articles, and briefs. **Figure 1** shows the flow chart of the literature selection process.

### Analysis method

This research employs GraphPad Prism for the analysis and visualization of annual paper counts, national publication trends, and proportions. Visualization analyses are carried out using CiteSpace, VOSviewer, and R software, while data management is conducted using Microsoft Excel 2019. CiteSpace, a document visualization tool created by Professor Chaomei Chen, is utilized to examine various indicators including countries/regions, authors, institutions, journals, and references (21). Additionally, CiteSpace is employed for keyword analysis to forecast research field trends.

This research employs GraphPad Prism for the analysis and visualization of the yearly publication count, national publication patterns, and proportions. The visualization analyses are carried out utilizing CiteSpace, VOSviewer, and R software, while data organization is executed through Microsoft Excel 2019. CiteSpace, created by Professor Chaomei Chen, is a tool for document visualization that examines various indicators including countries/regions, authors, institutions, journals, and references. In addition to its application in analyzing keywords for trend prediction in research fields, CiteSpace is utilized for constructing and visualizing bibliometric networks. VOSviewer, on the other hand, is a software tool specifically designed for the analysis of bibliometric networks, enabling the creation of visual network maps that offer a nuanced and thorough insight into the structure and evolution of scientific research (22).

### Bibliometric indicators

To comprehensively analyze the development trends and influence of the research field, this study employed various bibliometric indicators, including publication count, citation frequency, co-citation analysis, keyword co-occurrence analysis, citation burst analysis, H-index, and author collaboration network analysis. Publication count reflects the level of research activity in the field, while citation frequency measures the academic impact of the literature. Co-citation analysis reveals the knowledge connections between related works, and keyword co-occurrence analysis helps identify research hotspots and emerging trends. Citation burst analysis highlights key breakthroughs and emerging topics in the field. The H-index is used to assess the academic influence of scholars or journals, while author collaboration network analysis shows the cooperation patterns among researchers. Through these indicators, this study aims to uncover the core literature, key issues, and academic collaboration networks in the field (23, 24).

## Results

### Publishing trend

The findings of the study indicate that between January 1, 2004, and December 31, 2023, the WoSCC database documented 2,968 medical articles pertaining to the utilization of nanotechnology in the management of HCC. Of these articles, 2,468 were classified as research articles (94.31%) and 500 as review articles (5.69%). The publications involved contributions from 83 countries and regions, 2,885 research institutions, and a total of 13,255 authors.

**Figure 2:**
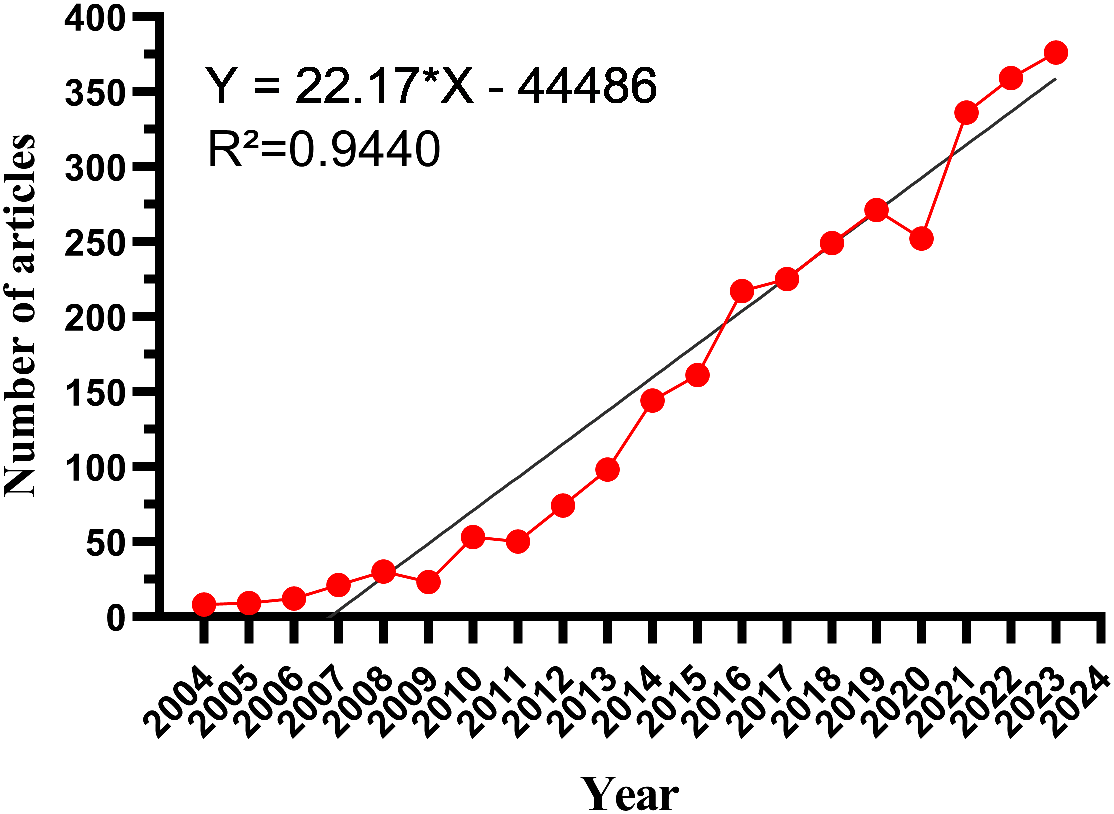
The trend in annual publication numbers on the application of nanotechnology in Hepatocellular Carcinoma diagnosis and treatment from 2004 to 2023.

The data presented indicate a discernible upward trajectory in the quantity of publications within this particular research domain over time. The evolution of research in this field can be delineated into three distinct phases: an initial phase spanning from 2004 to 2009 characterized by a modest output of publications not surpassing 50 annually, suggestive of limited scholarly interest. Subsequently, the second phase spanning from 2010 to 2015 witnessed a substantial surge in publications, emblematic of a period of accelerated advancement within the field. The third phase, spanning from 2016 to 2023, demonstrated sustained scholarly activity culminating in a peak in publications in 2023.The temporal progression depicted in **Figure 2** provides a clear illustration of the evolution and expansion of research hotspots in the field of nanotechnology applications for HCC diagnosis and treatment, reflecting the research activity in this area.

### Distribution of countries/regions and institutions

**Figure 3:**
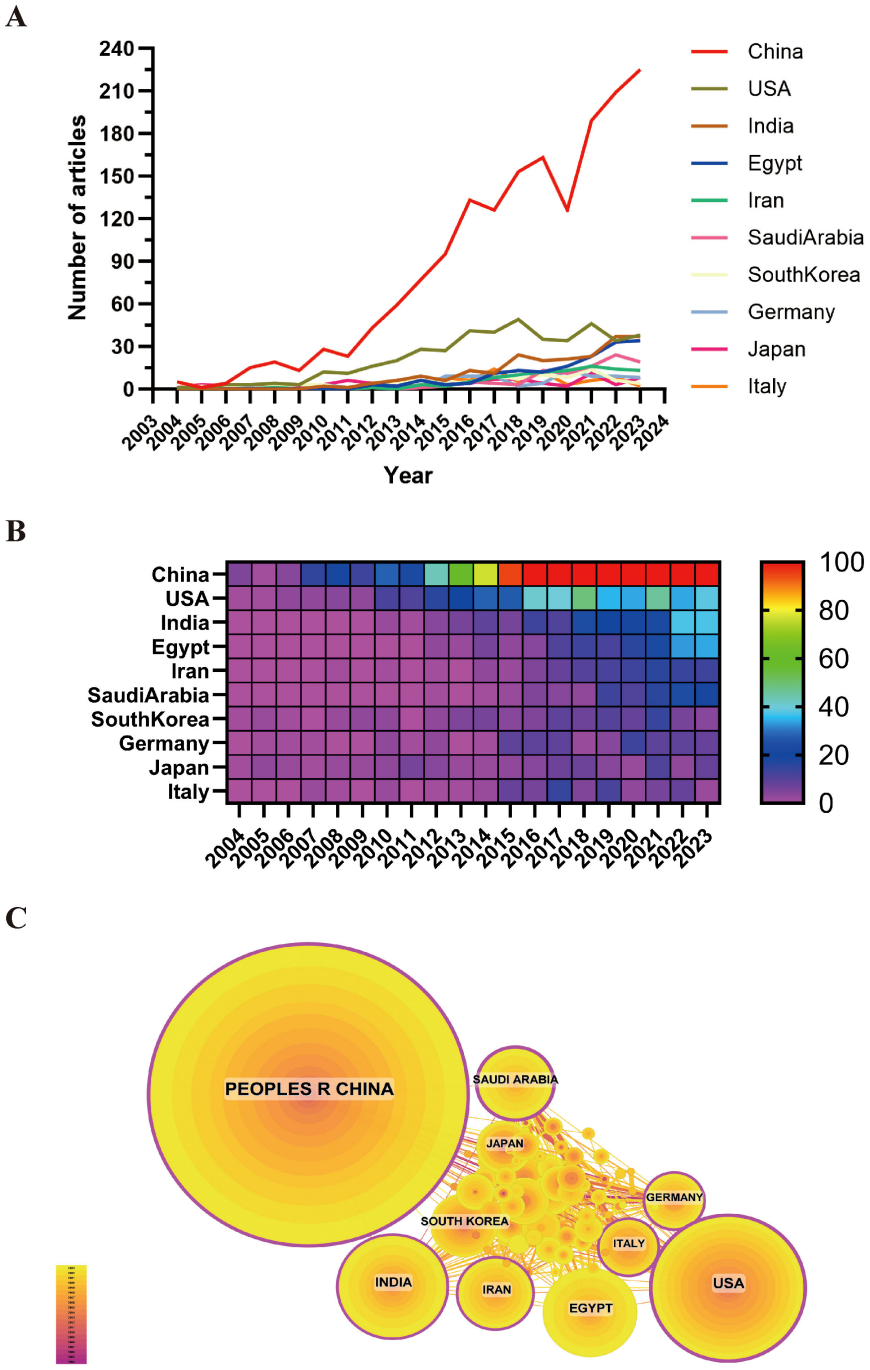
Over the past 20 years, the volume of publications and collaboration scenarios on the use of nanotechnology in Hepatocellular Carcinoma diagnosis and treatment across different countries. **(A)** Publication volume line chart; **(B)** Heat map of publication volume; **(C)** International collaboration network map.

Throughout the study period, 54 countries and regions globally engaged in medical research regarding the utilization of nanotechnology in the treatment of HCC. As evidenced by **Figures 3A, B**, the leading countries in terms of publication volume over the previous two decades were identified as China, the USA, India, Egypt, and Saudi Arabia, with China emerging as the frontrunner. China’s publication output of 1,700 papers represented 57.28% of the total, a notably higher proportion compared to other nations.

Chinese papers were cited 50,939 times, ranking first among all countries in terms of citation frequency (refer to **Supplementary Table S1**). Nevertheless, the citation-to-publication ratio for Chinese papers stands at 29.96, placing them seventh. This suggests that despite China’s significant contribution to research on the application of nanotechnology in HCC, the average quality of its publications is marginally lower than that of other nations. The United States is ranked second in terms of the number of publications, with a total of 445, and citations received, totaling 20,470. Additionally, the citation-to-publication ratio for U.S. papers is 45.90, suggesting a relatively high quality of research output.

In the context of collaboration networks, **Figure 3C** illustrates a significant cooperative partnership between China and the United States, both of which exhibit the highest publication outputs. Furthermore, China demonstrates robust collaborative ties with Japan, Saudi Arabia, India, and South Korea, whereas the United States shows closer collaborations with Italy, Germany, and Egypt.

**Figure 4:**
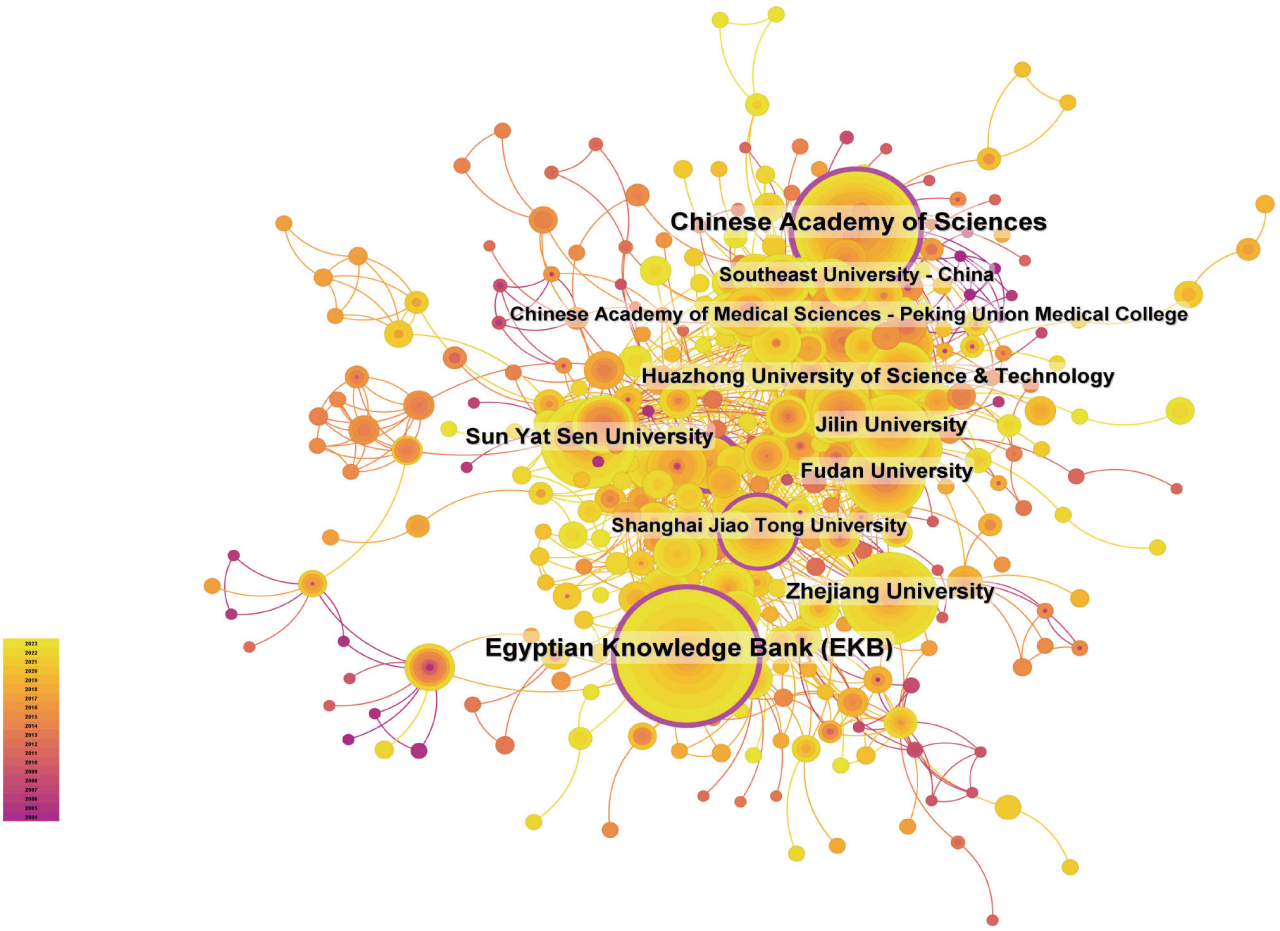
Network diagram illustrating institutional collaboration on the application of nanotechnology in Hepatocellular Carcinoma diagnosis and treatment.

A total of 2,885 institutions were found to have systematically published medical articles on the utilization of nanotechnology in HCC. Among the top ten institutions in terms of publication volume, nine are based in China, with the remaining one located in Egypt (refer to **Supplementary Table S2**, **Figure 4**). The Chinese Academy of Sciences emerged as the leading institution with 162 papers and 6,735 citations, resulting in an average of 41.57 citations per paper. The Egyptian Knowledge Bank (EKB) closely followed with 160 papers and 3,434 citations, averaging 21.47 citations per paper. Zhejiang University and Sun Yat-sen University are positioned third and fourth in terms of publication output, with 87 and 80 papers, respectively, indicating their significant research productivity and influence.

Through additional examination, it was determined that domestic institutions typically engage in collaboration with other units within their own nation, with international cooperation being comparatively restricted. As a result, we advocate for enhancing international institutional collaboration in order to address academic obstacles and facilitate the dissemination of global knowledge and technological progress.

### Journals and co-cited journals

**Figure 5:**
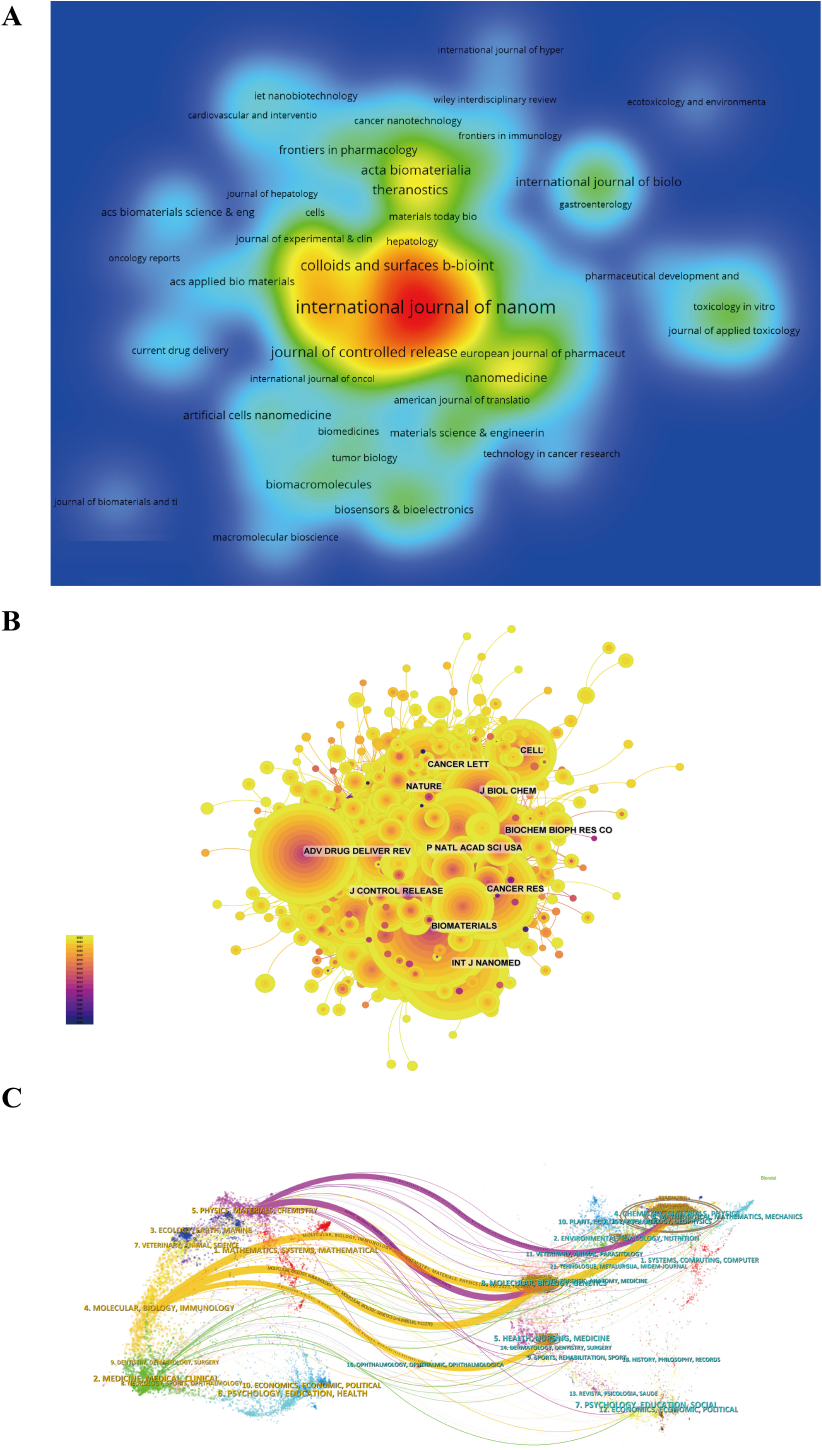
Information on journals publishing research on the application of nanotechnology in Hepatocellular Carcinoma diagnosis and treatment and their co-cited journal data. **(A)** Journal publication density map; **(B)** Journal co-citation network map; **(C)** Journal dual overlay map (Left: citing journals. Right: cited journals.).

**Figure 5A**, **Supplementary Tables S3** and **S4** present data on the top ten journals in terms of article publication and citation frequencies. The International Journal of Nanomedicine holds the highest rank with 180 articles, representing 6.06% of total publications, followed by Biomaterials (94 articles, 3.17%), International Journal of Pharmaceutics (85 articles, 2.86%), and Journal of Biomedical Nanotechnology (69 articles, 2.32%). Biomaterials boasts the highest impact factor (IF) of 14.0 among these high-output journals, with 90% of the journals falling within the Q1 or Q2 quartiles.

The academic influence of journals can be assessed by their co-citation frequency, which reflects the level of impact within the scientific community. Analysis of **Figure 5B** and **Supplementary Table S4** reveals that Biomaterials is the most frequently co-cited journal (1,710 citations), followed by the Journal of Controlled Release (1,567 citations) and the International Journal of Nanomedicine (1,256 citations). Among the top ten frequently co-cited journals, ACS Nano stands out with 1,122 citations and the highest impact factor of 17.1. It is noteworthy that all of these frequently co-cited journals are classified in the Q1/Q2 quartiles.

Dual-map overlays are utilized as a visualization tool to demonstrate interdisciplinary research focuses, citation pathways, and shifts in trends. This illustrative method highlights the discrepancy in journal citation patterns between the cited and citing journals. For instance, **Figure 5C** demonstrates that research published in molecular/biology/immunology journals is predominantly cited by research published in molecular/biology/genetics, health/nursing/medicine, and chemistry/materials/physics journals. Similarly, research published in journals within the physics/materials/chemistry fields is primarily cited by research published in journals within the molecular/biology/genetics and chemistry/materials/physics fields. This view can help researchers understand the interactions between different disciplines and the evolution of their academic influence.

### Authors and co-cited authors

**Figure 6:**
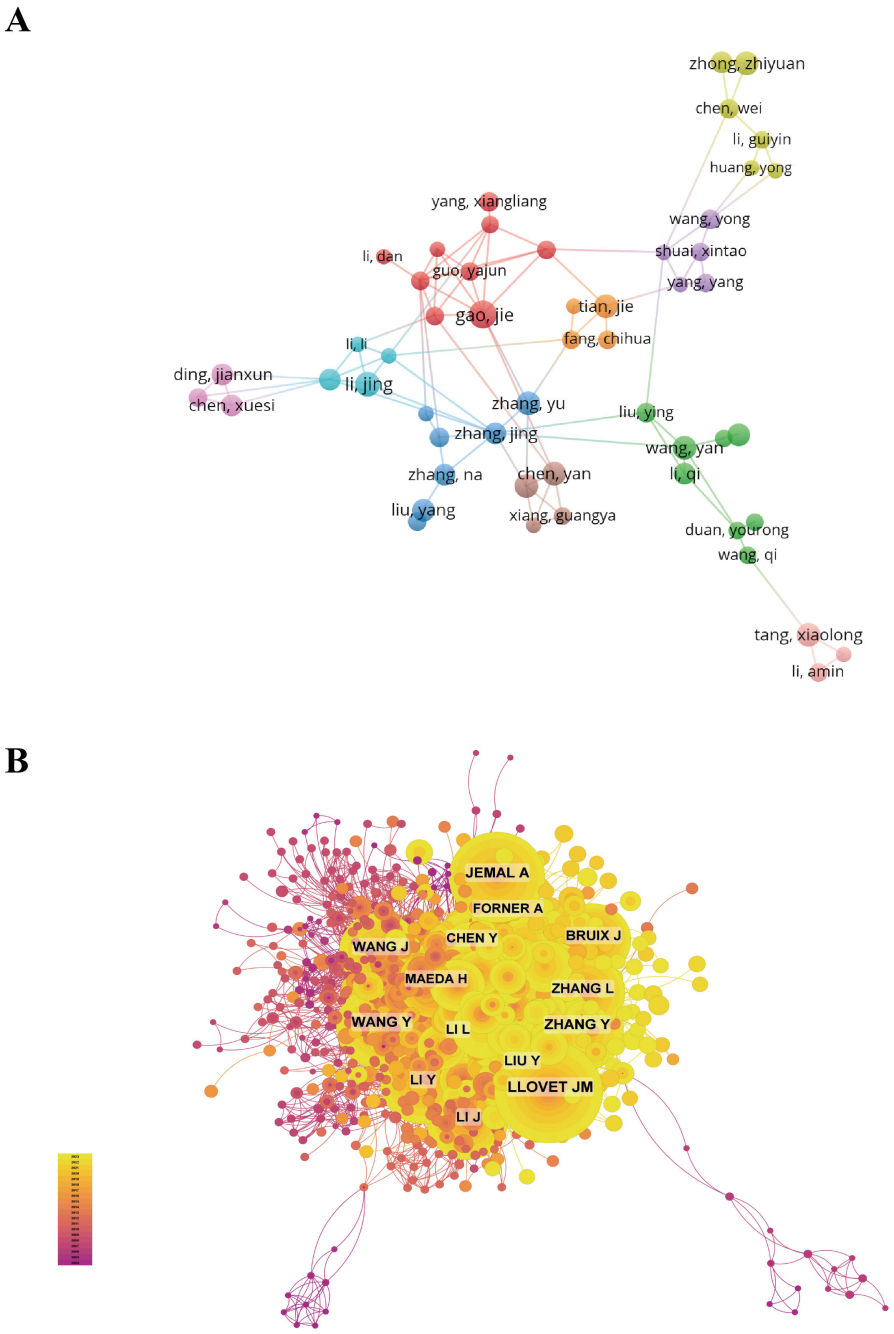
Author collaboration network map and author co-citation network map. **(A)** Author collaboration network map; **(B)** Author co-citation network map.

**Supplementary Table S5** presents the top 10 authors in the field of medical literature pertaining to the utilization of nanotechnology in HCC, with a collective publication count of 157 papers, representing 5.26% of all publications in this area. Gao Jie and Li Yan are the most prolific authors with 18 publications each, followed by Li Jing with 16 publications. It is noteworthy that all of the top 10 authors are affiliated with institutions in China. The network among these authors is visualized using CiteSpace (**Figure 6A**).

**Figure 6B** and **Supplementary Table S5** present the top 10 authors based on co-citation and citation frequency, respectively. A total of 54 authors have received more than 20 citations, suggesting a high level of prestige and influence in their research field. The largest nodes in the network are linked to the most frequently co-cited authors, such as Llovet JM (357 citations), Jemal A (275 citations), and Zhang Y (273 citations).

### Co-cited references and references burst

**Figure 7:**
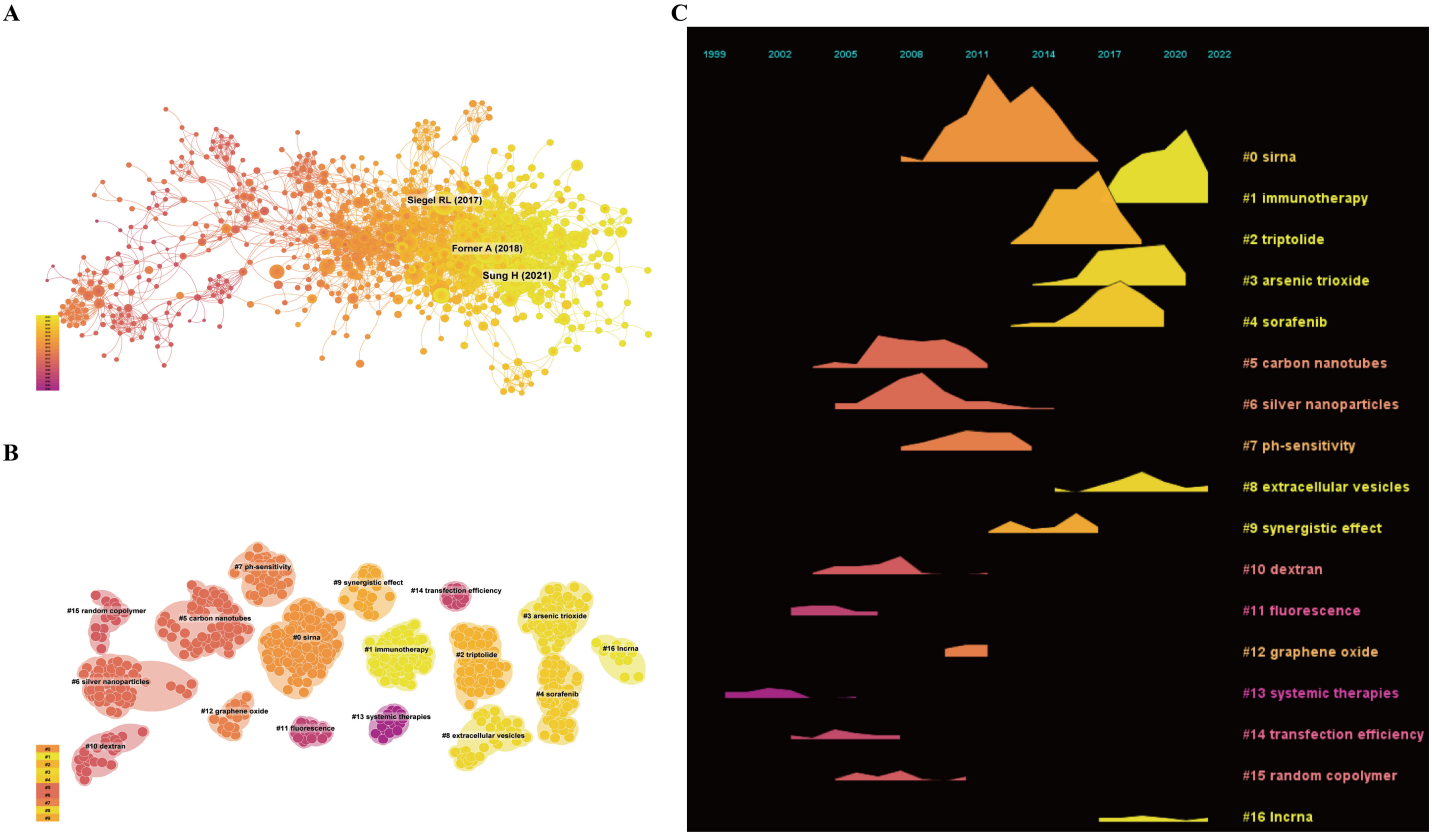
Co-cited reference information. **(A)** Co-cited literature network map; **(B)** Co-cited literature clustering map; **(C)** Co-cited literature volcano plot.

From 2004 to 2023, a co-citation network consisting of 1,169 nodes and 4,476 links was observed (as illustrated in **Figure 7A**). The top 10 most commonly co-cited articles are detailed in **Supplementary Table S6**, with the first and second positions occupied by publications from CA: A Cancer Journal for Clinicians titled “Global cancer statistics 2020: GLOBOCAN estimates of incidence and mortality worldwide for 36 cancers in 185 countries” and “Cancer Statistics, 2009”.These studies offer current data on recent cancer diagnoses and fatalities, consolidating information on cancer occurrence, death rates, and survival percentages (1, 25). They serve as a vital basis for developing and executing cancer management tactics, playing a significant role in enhancing cancer interventions on a global and regional scale. The article titled “Cancer nanomedicine: progress, challenges and opportunities” from Nature Reviews Cancer holds the seventh position in the ranking. It provides a comprehensive overview of the advancements, obstacles, and potential prospects in the realm of cancer nanomedicine (26). The article underscores the substantial promise of nanotechnology in enhancing the accuracy and effectiveness of cancer therapy, with a particular focus on minimizing adverse effects and augmenting drug potency via targeted drug delivery mechanisms (26). Nevertheless, despite numerous achievements in preclinical studies, the clinical implementation of nanomedicines is hindered by various technical and regulatory challenges. The article underscores the importance of resolving critical issues related to the commercialization and clinical adoption of these nanomedicines, including production expenses, scalability of manufacturing processes, and the assurance of sustained safety and effectiveness. The article titled “Challenges in liver cancer and possible treatment approaches” from Biochimica et Biophysica Acta - Reviews on Cancer, ranked tenth, delves into the obstacles encountered in the treatment of liver cancer and proposes potential solutions through interdisciplinary collaboration and technological innovation. Specifically, the article highlights the recent advancements in nanotechnology for the diagnosis and treatment of liver cancer (5).

Additionally, we performed co-citation reference clustering and timeline clustering analysis, as illustrated in **Figures 7B, C**. We found that fluorescence (cluster 11), systemic therapies (cluster 13), and transfection efficiency (cluster 14) were early research hotspots. Carbon nanotubes (cluster 5), silver nanoparticles (cluster 6), pH-sensitivity (cluster 7), synergistic effect (cluster 9), dextran (cluster 10), graphene oxide (cluster 12), and random copolymer (cluster 15) were mid-term research hotspots. siRNA (cluster 0), immunotherapy (cluster 1), triptolide (cluster 2), arsenic trioxide (cluster 4), extracellular vesicles (cluster 8), and lncRNA (cluster 16) are current hotspots and trends in the field.

### Keyword visualization analysis

**Figure 8:**
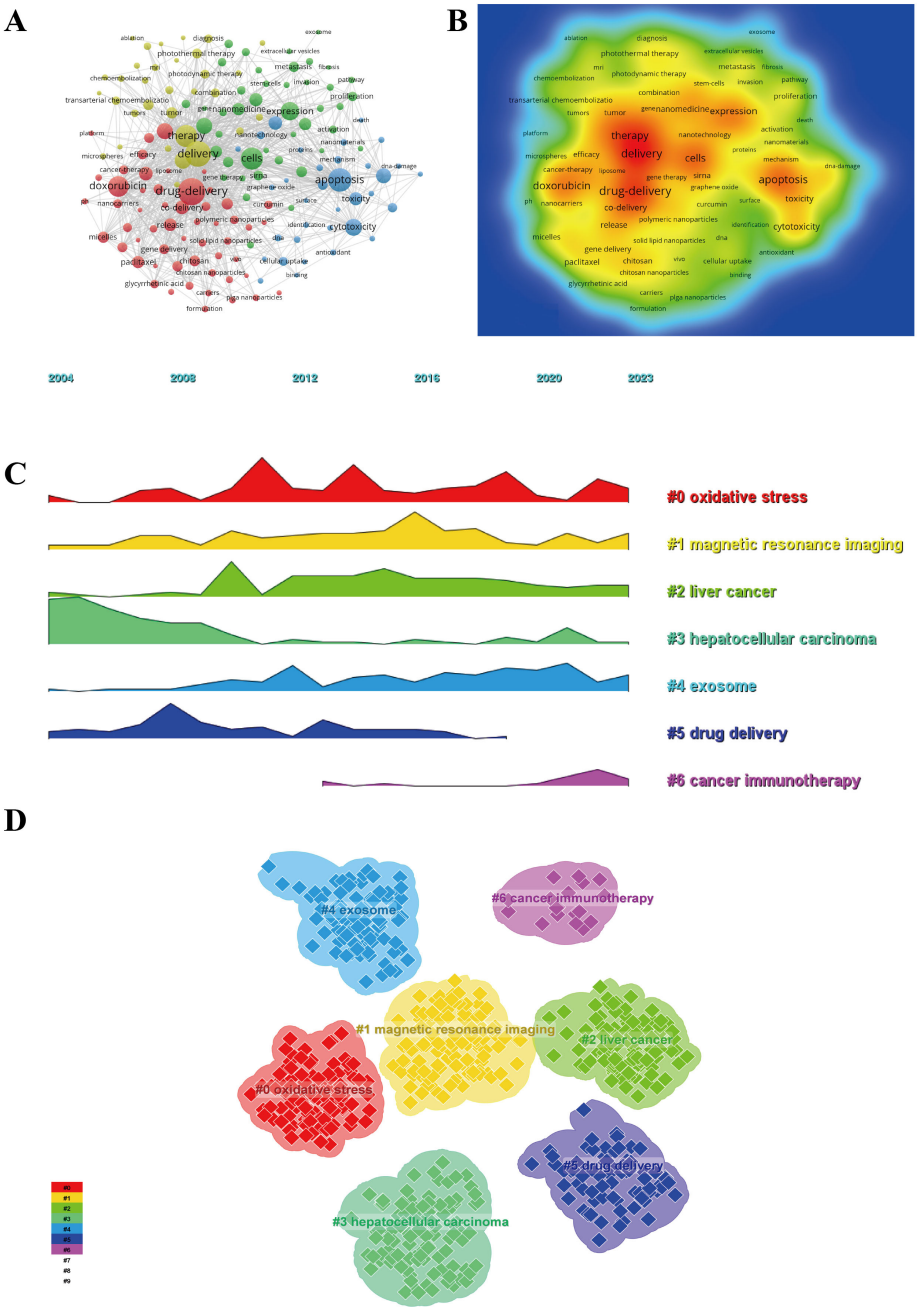
Keyword analysis of articles on the application of nanotechnology in hepatocellular carcinoma diagnosis and treatment. **(A)** High-frequency keywords network map; **(B)** Keyword density map; **(C)** Keyword clustering volcano plot; **(D)** Keyword clustering map.

Through keyword analysis, researchers can efficiently understand the present status and emerging trends within a particular academic discipline. Utilizing VOSviewer software for co-occurrence analysis, the most frequently appearing terms include “drug-delivery” (465 occurrences), “delivery” (428 occurrences), “apoptosis” (362 occurrences), “therapy” (331 occurrences), and “cells” (330 occurrences) as illustrated in **Supplementary Table S7** and **Figures 8A, B**. Insignificant keywords were removed, resulting in a network of 163 keywords that appeared a minimum of 29 times, leading to the formation of four distinct clusters. The initial cluster (red) consists of 54 keywords, including drug delivery, co-delivery, doxorubicin, release, chitosan, micelles, nanocarriers, efficacy, target delivery, bioavailability, cisplatin, design, formulation, system, receptor, stability, pH, and liposome. The second cluster, denoted by the color green, comprises 43 keywords such as expression, cells, sorafenib, breast cancer, metastasis, invasion, activation, angiogenesis, autophagy, cell death, exosome, gene therapy, inflammation, immunotherapy, pathway, and stem cell. The third cluster, represented by the color blue, encompasses 34 keywords including apoptosis, cytotoxicity, oxidative stress, reactive oxygen species, silver nanoparticles, antioxidant, DNA, induction, mechanism, nanomaterial, protein, surface, and transfection. In contrast, the fourth cluster, denoted by the color yellow, comprises 32 keywords associated with tumor, therapy, delivery, model, antibody, agents, combination, diagnosis, MRI, particles, peptide, radiotherapy, survival, therapy, and quantum dot. Furthermore, the utilization of CiteSpace software facilitated the generation of volcano plots, which offer a visual representation of research hotspots and their evolution over time (refer to **Figures 8C, D**). Such analyses contribute to a comprehensive understanding of the predominant themes and potential trajectories within the field of study.

### Co-cited references and keywords

**Figure 9:**
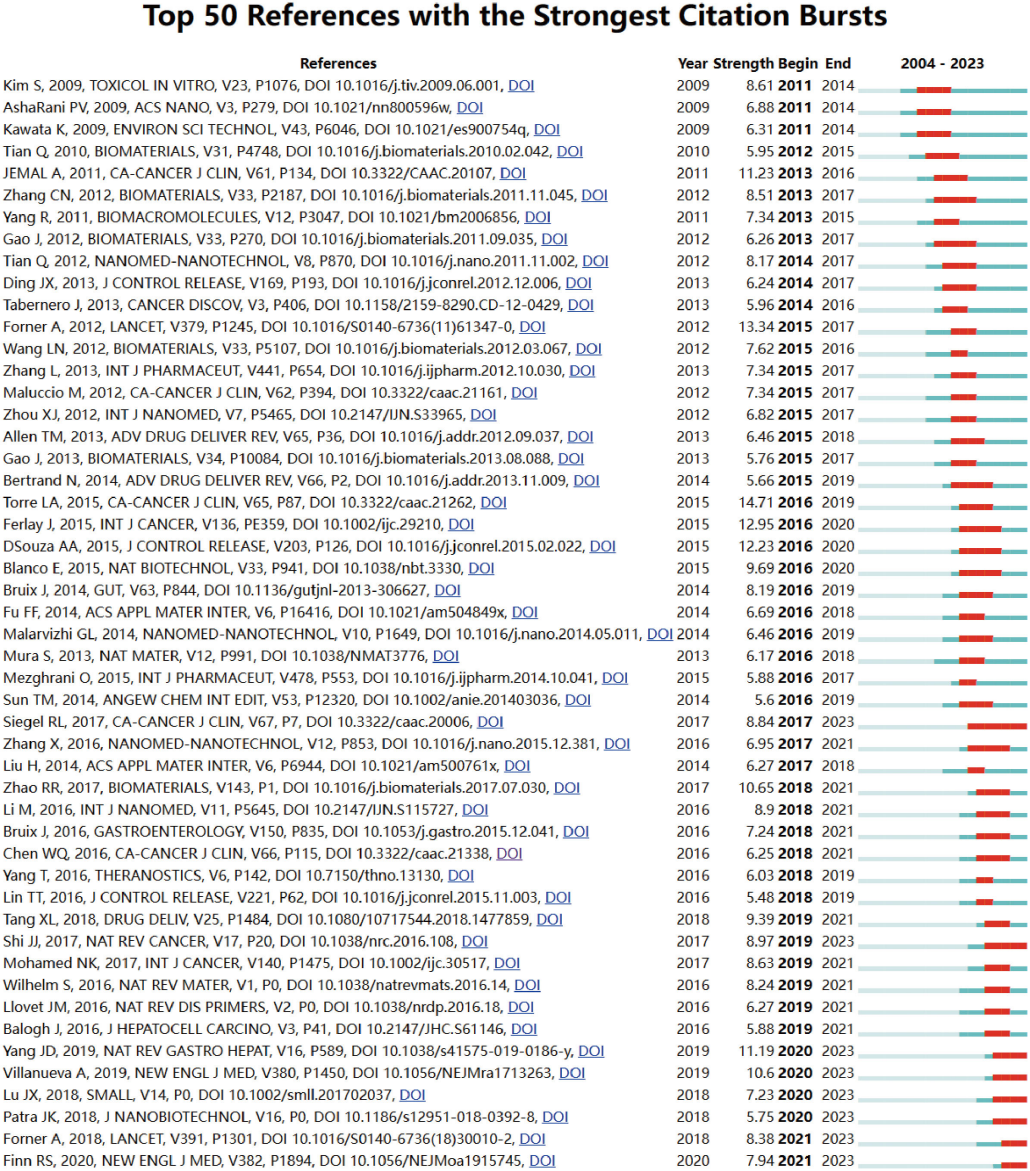
Top 50 references with the strongest citation bursts.

Through the utilization of CiteSpace analysis, we have discerned the 50 most impactful citation burst papers within the realm of nanotechnology applications in HCC, as depicted in **Figure 9**. These papers have been categorized into three distinct groups. The initial category encompasses publications that present worldwide cancer epidemiology information, exemplified by the most prominent citation burst reference in the figure (14.71), which is the article “Global cancer statistics, 2012” published in CA: A Cancer Journal for Clinicians. This article offers an in-depth analysis of cancer incidence and mortality on a global scale, examining the variations in geographic and gender distribution across different types of cancer. The primary objective of the article is to increase awareness of the significant burden of cancer worldwide and underscore the critical role of prevention and screening in mitigating cancer rates. Through comparative analysis of data from diverse countries and regions, the article underscores the pivotal role of cancer control strategies in promoting global health (27). The second category comprises articles that offer comprehensive analyses of the epidemiology, etiology, diagnosis, and treatment approaches for HCC. For example, the publication with the second highest citation burst reference (13.37) in the figure is a study in Lancet titled “Hepatocellular carcinoma.” This article extensively examines diverse risk factors associated with hepatocellular carcinoma, such as hepatitis B and C virus infections, alcoholic liver disease, non-alcoholic fatty liver disease (NAFLD), and its correlation with obesity and diabetes. Moreover, the article delves into strategies for early detection of HCC through the utilization of imaging techniques and biomarkers. It also discusses the existing treatment modalities, including surgical resection, local ablation, liver transplantation, and systemic therapies, encompassing the most recent advancements in targeted medications and immunotherapies. The article underscores the significance of tailored treatment regimens and outlines potential avenues for future research (28). The third category encompasses research that offers comprehensive examinations of the significant functions of nanotechnology in targeted drug delivery systems for hepatocytes, immunotherapy, gene therapy, diagnostics and imaging, and multimodal treatments. For instance, the publication with the fourth highest citation burst reference (12.23) in the figure is found in the Journal of Controlled Release, entitled “Asialoglycoprotein receptor mediated hepatocyte targeting - strategies and applications.” This study focuses on the development of various nanoparticles designed to be selectively recognized by ASGPR, leading to effective targeting of hepatocytes. The nanoparticles were able to increase their binding affinity with ASGPR in the liver by undergoing surface modifications, such as the attachment of specific ligands or antibodies. This facilitated direct drug delivery to hepatocytes, resulting in a notable enhancement of therapeutic efficacy and a reduction in potential toxicity to non-target organs. The article provides a comprehensive examination of the utilization of this nanotechnology in the management of liver cancer and other liver-related disorders, including an exploration of the technical obstacles and potential avenues for future research (29). All 50 articles were published within the timeframe of 2004 to 2023, showcasing their enduring impact over the previous two decades. It is noteworthy that eight of these articles are presently undergoing a surge in citations, underscoring the ongoing interest in the medical utilization of nanotechnology for the treatment of HCC.

**Figure 10:**
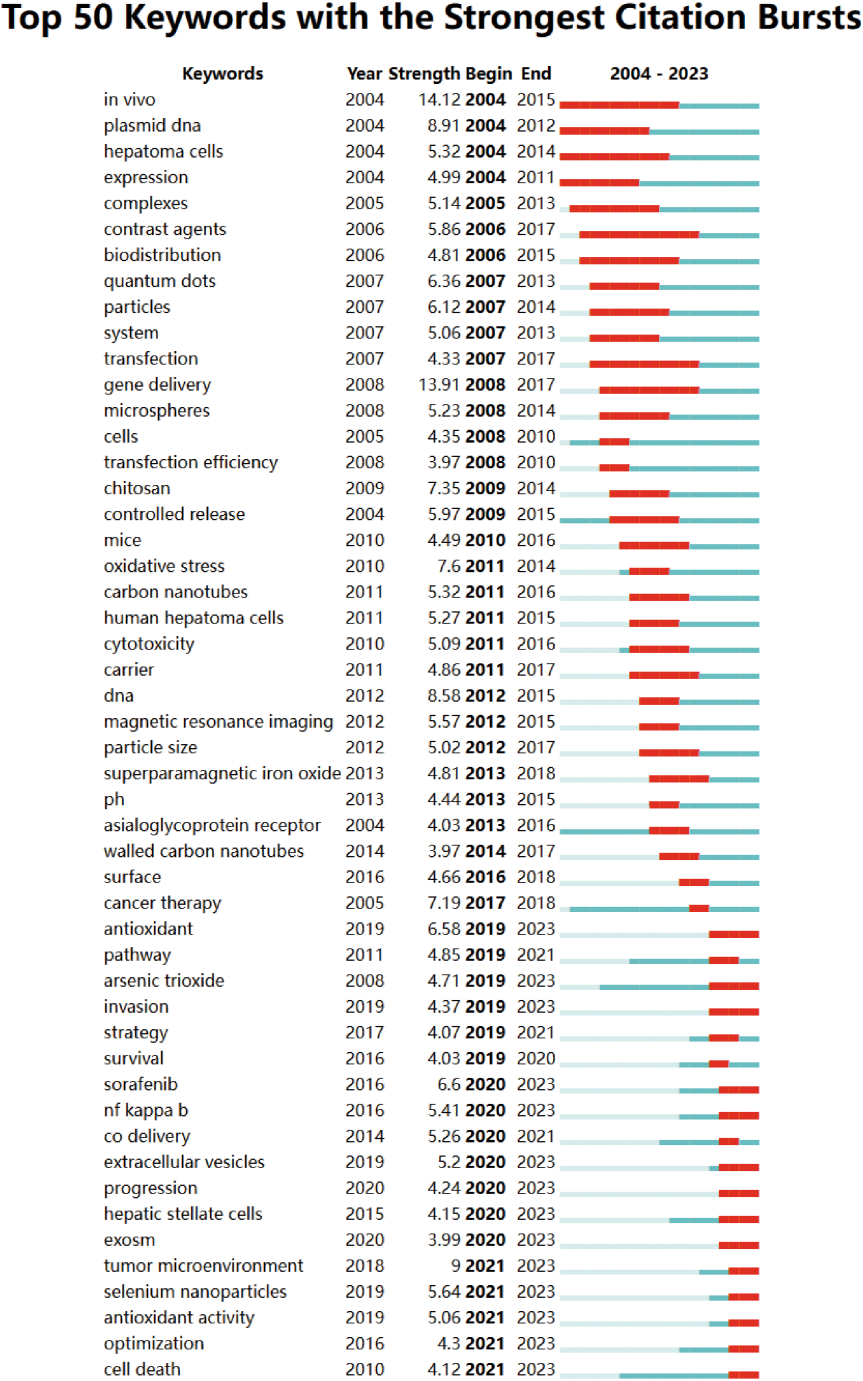
Top 50 keywords with the strongest citation bursts.

Furthermore, among the 756 key burst keywords, our attention was directed towards the 50 most closely associated keywords, as depicted in **Figure 10**. These keywords serve to illuminate the prevailing research trends and potential avenues for future exploration within the field. The findings of this analysis offer valuable insights into the utilization of nanomedicine for the treatment of HCC, as well as suggest promising directions for further investigation.

## Discussion

Bibliometrics is an academic discipline that examines the distribution, patterns, and quantitative relationships within scientific literature and information (17). Utilizing quantitative methods, bibliometrics uncovers developmental trends and fundamental structures within research fields (16). The analytical tools and theoretical models employed in this field are highly valuable for facilitating the rational organization and effective management of scientific research, thereby enhancing the data-driven and precise nature of research decision-making processes. In this study, we chose to use the Web of Science (WOS) database because it comprehensively covers high-quality academic journals and has a robust citation index, making it highly suitable for bibliometric analysis. Furthermore, WOS provides reliable and carefully curated data, which helps track research trends, making it the preferred database for our investigation into the application of nanotechnology in liver cancer treatment (30, 31). This research study examines the utilization of nanotechnology in the detection and management of liver cancer, conducting a comprehensive analysis of 2,968 scholarly articles released from January 1, 2004, to December 31, 2023. These articles encompass contributions from 83 countries and regions, as well as 2,885 research institutions, and were disseminated across 588 academic publications, illustrating the widespread and profound international research cooperation within this domain. The findings indicate a rising interest in the study of nanotechnology’s role in the diagnosis and treatment of HCC, underscoring its increasing significance within the global research landscape.

The quantity of scholarly publications is frequently regarded as a crucial metric for tracking advancements within a specific discipline (32). In the last two decades, there has been a notable surge in the volume of medical research papers focused on the utilization of nanotechnology for diagnosing and treating HCC, underscoring the escalating research attention and advancements in this domain. Between 2004 and 2009, there was a limited amount of scholarly literature focusing on the utilization of nanotechnology in the treatment of HCC, with an average of slightly more than ten publications per year. During this timeframe, the field had not garnered significant interest from the academic community. However, with advancements in technology and the growing need for effective liver cancer therapies, the annual publication output stabilized at over 100 from 2010 to 2015.The consistent increase in publication output during this period suggests a growing interest among researchers in the field, resulting in a substantial volume of both theoretical and practical research. Since 2016, the annual number of publications in this area has surpassed 200, indicating a more accelerated growth trajectory. The utilization of nanotechnology in the diagnosis and treatment of HCC has emerged as a focal point of research activity. The observed growth trend serves to illustrate the potential of nanotechnology in medical applications and also signifies the growing global research interest in addressing the significant health concern of HCC (7).

The quantity and quality of publications serve as significant measures of a country’s research output and the performance of its institutions. As evidenced by **Figures 3A, B**, China, the USA, India, Egypt, and Saudi Arabia emerge as the top five countries in terms of publication volume within this field. Notably, China leads with 1,700 published papers, accounting for 57.28% of the total, and garnering 50,939 citations. However, China’s citation-to-publication ratio stands at 29.96, positioning it in seventh place. China’s prominence in the field of nanotechnology applications in HCC diagnosis and treatment is underscored by its centrality score of 0.31, positioning it as a global leader in research on this subject. In comparison, the United States ranks second with 445 published papers and 20,470 citations, also securing the second position in terms of citation count. The United States’ citation-to-publication ratio of 45.9 places it in third position. These findings suggest that both China and the United States possess notable strengths in the realm of nanotechnology applications for HCC diagnosis and treatment. China’s prominent position in publication volume underscores its prolific output, yet its comparatively lower citation rate implies potential for enhancement in terms of international influence and academic recognition. Conversely, the United States, ranking second in publication volume, exhibits a high citation rate and citation-to-publication ratio, indicating superior quality and extensive global recognition of its research. Research originating from the United States not only boasts substantial quantity but also excels in quality and impact, with its findings being highly esteemed and widely implemented on a global scale. In the realm of nanotechnology applications for the diagnosis and treatment of HCC, both China and the United States are prominent nations. The global research collaboration network, as depicted in **Figure 3C**, illustrates the intricate patterns and interactions of scientific cooperation among various countries (33). Through an analysis of this collaboration network, several noteworthy characteristics emerge. Primarily, the United States and China, as frontrunners in this research domain, have forged extensive collaborative ties with one another and with numerous other nations. This international collaboration facilitates the exchange of scientific resources and knowledge, as well as expedites the advancement and implementation of nanotechnology in the treatment of HCC. Additionally, Asian nations, particularly China, Japan, and South Korea, are pivotal contributors to this network, engaging in both regional partnerships and maintaining strong scientific connections with European and North American countries, thereby showcasing their significant involvement and impact in worldwide scientific collaboration. Upon examination of the worldwide research output regarding the utilization of nanomedicine for the treatment of HCC, it is evident that Chinese institutions play a prominent role in this field. The Chinese Academy of Sciences, in particular, stands out due to its significant number of published papers and noteworthy citation counts, underscoring its substantial influence and scholarly contributions to this particular area of research. Despite the commendable performance of Chinese institutions in terms of both quantity and quality, it is important to acknowledge the limited collaboration between domestic and international institutions. This lack of collaboration may impede the dissemination of knowledge on a global scale and hinder the full potential of innovation. Therefore, we advocate for increased cooperation between domestic and international institutions. Collaborative efforts across borders have the potential to dismantle academic barriers, facilitate knowledge exchange, expedite technological advancements, and enhance the international reach and impact of research endeavors (34).

Examining the attributes of international peer-reviewed journals is essential for comprehending contemporary research patterns, aiding scholars in making well-informed choices when selecting journals for disseminating their research outcomes (35). This investigation reveals that, of the top ten journals with the most publications, eight are classified under the JCR Q1 category. Notably, the International Journal of Nanomedicine ranks highest in publication volume for the utilization of nanotechnology in liver cancer treatment, while Biomaterials is distinguished for its notable impact factor (IF).Co-citation is the practice of citing two or more academic journals together in scientific documents, providing insight into the knowledge framework and research patterns within a specific academic discipline. In the present investigation, Biomaterials emerges as the predominant journal with 1,710 co-citations, establishing itself as the central publication in this domain by ranking second in publication frequency and first in co-citations. For researchers focused on the utilization of nanomedicine in HCC studies, comprehending the attributes and trajectories of these leading journals is imperative for advancing their scholarly pursuits.

When examining the scholarly impact of nanotechnology on liver cancer therapy, it is crucial to recognize the leading researchers in this area. A review of **Supplementary Table S5** reveals that Gao Jie, Li Yan, and Li Jing are the foremost authors with the greatest volume of publications, underscoring their significant research output and influence in this domain. LLOVET JM, JEMAL A, and ZHANG Y emerge as the three most frequently co-cited authors, suggesting widespread academic acknowledgment of their research and underscoring their substantial contributions and central influence in the utilization of nanotechnology for the treatment of HCC. LLOVET JM, being the most frequently cited author in the field, is presumed to have established the research trajectory and standards, thereby exerting a significant influence on the advancement and emphasis of subsequent studies. The elevated citation rate attributed to his work not only underscores its exceptional quality and originality, but also signifies the substantial value of his findings in both clinical practice and scientific discourse. It is significant to note that while the top ten authors in publication volume are exclusively from China, signifying the rapid advancement of research in this field within the country, Chinese authors are less represented among the most cited authors. This indicates that despite the growth in quantity, there remains a need for enhancement in the quality and global impact of Chinese research in this particular field. This observation underscores the necessity of improving research standards and fostering international partnerships to elevate China’s standing and influence within the worldwide scientific community.

Through the examination of the co-citation network map, clustering map, and volcano plot in this research, key academic articles and research trends within this field are identified. The analysis involves 1,169 nodes and 4,476 links, illustrating the extensive connections and profound exchanges within the research domain. Notably, the most frequently co-cited literature relates to global statistics, epidemiological characteristics, and emerging treatment strategies for HCC. These foundational studies provide important context for the application of nanotechnology in liver cancer treatment, laying a solid groundwork for further research. With the continuous innovation in HCC treatment strategies, nanotechnology—especially advancements in drug delivery mechanisms—has shown significant promise.

By employing time-series clustering analysis, the progression of nanotechnology applications in HCC diagnosis and treatment can be discerned. This analysis reveals a shift from fundamental research to practical clinical implementation, with a growing focus on enhancing the effectiveness and safety of therapies. This transition underscores how nanotechnology is not only contributing to the early diagnosis of HCC but also improving the therapeutic outcomes of existing treatment methods, particularly by advancing drug delivery systems (7, 36). These systems demonstrate potential for improving drug targeting, controlling release rates, and reducing drug toxicity, thus offering exciting possibilities for more effective HCC therapies. These findings suggest that the potential of nanotechnology in liver cancer treatment is expanding, offering valuable insights for both clinical applications and future research directions (37).

In addition, the co-occurrence analysis of keywords conducted with VOSviewer highlights the evolving research focal points within the field of nanotechnology for HCC treatment. Keywords such as “drug-delivery” and “apoptosis” emphasize the central role of drug delivery systems in HCC therapy, reflecting a strong research emphasis on enhancing these mechanisms to improve therapeutic efficacy while minimizing adverse effects. The clustering of keywords reveals the broad range of applications of nanotechnology, including advancements in drug delivery systems, nanocarriers, investigations into cell death mechanisms, oxidative stress responses, and the development of diagnostic and therapeutic strategies (38). These applications underscore nanotechnology’s promising role in precision medicine, diagnostics, and its ability to unravel HCC pathology, as well as innovate treatment approaches.

Furthermore, the integration of nanotechnology and tumor biology through interdisciplinary collaboration is expected to play a crucial role in advancing this field. By combining insights from both disciplines, further progress can be made in refining nanomedicine technologies, ultimately improving the precision and effectiveness of cancer therapies. This collaboration will not only enhance the efficacy of current treatments but also contribute to developing personalized and innovative therapeutic solutions for HCC (39).

These trends and insights provide valuable guidance for future research efforts, helping to establish a strong scientific foundation for clinical applications. The ongoing developments in nanotechnology, particularly in the context of HCC therapy, indicate that this field holds vast potential for improving both early diagnosis and the effectiveness of treatment methods. Researchers should continue to focus on optimizing existing drug delivery systems and exploring new nanotechnology-based therapeutic strategies. Moreover, international collaboration will be crucial in accelerating these advancements, ensuring the broader global impact of this research.

In conclusion, the role of nanotechnology in liver cancer treatment is not only advancing the understanding of HCC but also paving the way for more effective and personalized treatment options. Future research should prioritize interdisciplinary collaboration and the optimization of therapeutic technologies to fully harness the potential of nanotechnology in the fight against HCC.

Our research offers numerous notable strengths. Primarily, it represents the inaugural comprehensive, unbiased, precise, and user-friendly analysis of the literature regarding the utilization of nanotechnology in the diagnosis and treatment of HCC, delving into the emerging patterns in this domain. Consequently, our results have the potential to offer comprehensive direction for scholars and healthcare practitioners specializing in this particular area. Furthermore, we utilized various bibliometric tools to conduct a comprehensive analysis of research trends from diverse viewpoints, thereby enriching the multidimensional understanding of the study. Nevertheless, it is important to acknowledge the constraints of the study. These limitations encompass the omission of pertinent literature from prominent databases like PubMed, Embase, and Ovid, as well as the focus solely on English-language publications, potentially leading to language bias. Consequently, while our analysis offers valuable perspectives, it may not encompass the entirety of research within this domain.

## Conclusions

This study conducted a comprehensive analysis of the literature concerning the utilization of nanotechnology in the diagnosis and treatment of HCC from 2004 to 2023. Utilizing bibliometric methods, the research effectively elucidated the present status and developing trends within this domain. These results offer valuable insights to researchers, aiding in the identification of established research priorities and emerging areas of interest, thereby facilitating the pursuit of novel research avenues in this field.

## Data Availability

The original contributions presented in the study are included in the article/**Supplementary Material**. Further inquiries can be directed to the corresponding authors.
