# Supplementary material for: Seeing beyond words: nanotechnology in hepatocellular carcinoma - a bibliometric study
Source: Front Oncol. 2025 Jan 15;14:1487198. doi: 10.3389/fonc.2024.1487198 (PMC11774701; doi:10.3389/fonc.2024.1487198)
Supplement: Supplementary file 1 [file Table1.docx]

Table S1: Publication output of the top 10 countries/regions in the study of nanotechnology applications for Hepatocellular Carcinoma diagnosis and treatment.

| Rank | Country/region | Article counts | centrality | Percentage (%) | Citation | Citation per publication |
| --- | --- | --- | --- | --- | --- | --- |
| 1 | CHINA | 1700 | 0.31 | 57.28% | 50939 | 29.96 |
| 2 | USA | 446 | 0.27 | 15.03% | 20470 | 45.90 |
| 3 | INDIA | 214 | 0.1 | 7.21% | 6958 | 32.51 |
| 4 | EGYPT | 162 | 0.09 | 5.46% | 3438 | 21.22 |
| 5 | SAUDI ARABIA | 99 | 0.21 | 3.34% | 2454 | 24.79 |
| 6 | IRAN | 99 | 0.13 | 3.34% | 2358 | 23.82 |
| 7 | SOUTH KOREA | 94 | 0.05 | 3.17% | 4621 | 49.16 |
| 8 | GERMANY | 83 | 0.12 | 2.80% | 2845 | 34.28 |
| 9 | JAPAN | 77 | 0 | 2.59% | 3575 | 46.43 |
| 10 | ITALY | 73 | 0.13 | 2.46% | 3061 | 41.93 |
