# Supplementary material for: Seeing beyond words: nanotechnology in hepatocellular carcinoma - a bibliometric study
Source: Front Oncol. 2025 Jan 15;14:1487198. doi: 10.3389/fonc.2024.1487198 (PMC11774701; doi:10.3389/fonc.2024.1487198)
Supplement: Supplementary file 2 [file Table2.docx]

Table S2: Publication output of the top 10 institutions in the study of nanotechnology applications for Hepatocellular Carcinoma diagnosis and treatment.

| Rank | Institution | Country | Number of studies | Total citations | Average citation |
| --- | --- | --- | --- | --- | --- |
| 1 | Chinese Academy of Sciences | China | 162 | 6735 | 41.57 |
| 2 | Egyptian Knowledge Bank (EKB) | EGYPT | 160 | 3434 | 21.46 |
| 3 | Zhejiang University | China | 87 | 2694 | 30.97 |
| 4 | Sun Yat Sen University | China | 80 | 2300 | 28.75 |
| 5 | Fudan University | China | 65 | 2375 | 36.54 |
| 6 | Huazhong University of Science & Technology | China | 61 | 1891 | 31.00 |
| 7 | Jilin University | China | 55 | 1985 | 36.09 |
| 8 | Shanghai Jiao Tong University | China | 52 | 1464 | 28.15 |
| 9 | Chinese Academy of Medical Sciences - Peking Union Medical College | China | 47 | 2013 | 42.83 |
| 10 | Southeast University - China | China | 43 | 748 | 17.40 |
