# Supplementary material for: Seeing beyond words: nanotechnology in hepatocellular carcinoma - a bibliometric study
Source: Front Oncol. 2025 Jan 15;14:1487198. doi: 10.3389/fonc.2024.1487198 (PMC11774701; doi:10.3389/fonc.2024.1487198)
Supplement: Supplementary file 3 [file Table3.docx]

Table S3: Data on publication volume within the top 10 journals dedicated to research on nanotechnology applications for Hepatocellular Carcinoma diagnosis and treatment.

| Rank | Journal | Article counts | Percentage（2968） | IF | Quartile in category |
| --- | --- | --- | --- | --- | --- |
| 1 | international journal of nanomedicine | 180 | 6.06% | 8.0 | Q2 |
| 2 | biomaterials | 94 | 3.17% | 14.0 | Q1 |
| 3 | international journal of pharmaceutics | 85 | 2.86% | 5.8 | Q1 |
| 4 | journal of biomedical nanotechnology | 69 | 2.32% | 2.9 | Q4 |
| 5 | journal of controlled release | 66 | 2.22% | 10.8 | Q1 |
| 6 | journal of materials chemistry b | 66 | 2.22% | 7.0 | Q1 |
| 7 | colloids and surfaces b-biointerfaces | 62 | 2.09% | 5.8 | Q1 |
| 8 | journal of nanobiotechnology | 50 | 1.68% | 10.2 | Q1 |
| 9 | theranostics | 50 | 1.68% | 12.4 | Q1 |
| 10 | acta biomaterialia | 48 | 1.62% | 9.7 | Q1 |
