# Supplementary material for: Seeing beyond words: nanotechnology in hepatocellular carcinoma - a bibliometric study
Source: Front Oncol. 2025 Jan 15;14:1487198. doi: 10.3389/fonc.2024.1487198 (PMC11774701; doi:10.3389/fonc.2024.1487198)
Supplement: Supplementary file 4 [file Table4.docx]

Table S4: Co-citation table of the top 10 journals in the study of nanotechnology applications for Hepatocellular Carcinoma diagnosis and treatment.

| Rank | Cited Journal | Co-Citation | IF（2022） | Quartile in category |
| --- | --- | --- | --- | --- |
| 1 | BIOMATERIALS | 1710 | 14.0 | Q1 |
| 2 | J CONTROL RELEASE | 1567 | 10.8 | Q1 |
| 3 | INT J NANOMED | 1256 | 8.0 | Q2 |
| 4 | ADV DRUG DELIVER REV | 1135 | 16.1 | Q1 |
| 5 | ACS NANO | 1122 | 17.1 | Q1 |
| 6 | CANCER RES | 1118 | 11.2 | Q1 |
| 7 | INT J PHARMACEUT | 1090 | 5.8 | Q1 |
| 8 | P NATL ACAD SCI USA | 946 | 11.1 | Q1 |
| 9 | PLOS ONE | 880 | 3.7 | Q2 |
| 10 | HEPATOLOGY | 848 | 14.0 | Q1 |
