# Supplementary material for: Seeing beyond words: nanotechnology in hepatocellular carcinoma - a bibliometric study
Source: Front Oncol. 2025 Jan 15;14:1487198. doi: 10.3389/fonc.2024.1487198 (PMC11774701; doi:10.3389/fonc.2024.1487198)
Supplement: Supplementary file 5 [file Table5.docx]

Table S5: Top 10 authors and co-cited authors related to the study of nanotechnology applications for Hepatocellular Carcinoma diagnosis and treatment.

| Rank | Author | Count | Location | Rank | Co-cited author | Citation |
| --- | --- | --- | --- | --- | --- | --- |
| 1 | gao, jie | 18 | China | 1 | LLOVET JM | 357 |
| 2 | li, yan | 18 | China | 2 | JEMAL A | 275 |
| 3 | li, jing | 16 | China | 3 | ZHANG Y | 273 |
| 4 | chen, yan | 15 | China | 4 | WANG Y | 248 |
| 5 | lee, robert j. | 15 | China | 5 | LIU Y | 244 |
| 6 | tang, xiaolong | 15 | China | 6 | LI Y | 231 |
| 7 | tian, jie | 15 | China | 7 | ZHANG L | 217 |
| 8 | wang, yan | 15 | China | 8 | WANG J | 204 |
| 9 | zhang, yu | 15 | China | 9 | BRUIX J | 197 |
| 10 | zhong, zhiyuan | 15 | China | 10 | LI J | 197 |
