# Supplementary material for: Seeing beyond words: nanotechnology in hepatocellular carcinoma - a bibliometric study
Source: Front Oncol. 2025 Jan 15;14:1487198. doi: 10.3389/fonc.2024.1487198 (PMC11774701; doi:10.3389/fonc.2024.1487198)
Supplement: Supplementary file 6 [file Table6.docx]

Table S6: Top 10 co-cited references related to nanotechnology applications for Hepatocellular Carcinoma diagnosis and treatment.

| Rank | Title | Journal | author(s) | Total citations |
| --- | --- | --- | --- | --- |
| 1 | Global cancer statistics 2020: GLOBOCAN estimates of incidence and mortality worldwide for 36 cancers in 185 countries | *CA-A CANCER JOURNAL FOR CLINICIANS* | Sung H | 103 |
| 2 | Cancer Statistics, 2009 | *CA-A CANCER JOURNAL FOR CLINICIANS* | Siegel RL | 65 |
| 3 | Hepatocellular carcinoma | *LANCET* | Forner A | 65 |
| 4 | A global view of hepatocellular carcinoma: trends, risk, prevention and management | *NATURE REVIEWS GASTROENTEROLOGY & HEPATOLOGY* | Yang JD | 58 |
| 5 | Hepatocellular Carcinoma | *NEW ENGLAND JOURNAL OF MEDICINE* | Villanueva A | 55 |
| 6 | Simultaneous inhibition of growth and metastasis of hepatocellular carcinoma by co-delivery of ursolic acid and sorafenib using lactobionic acid modified and pH-sensitive chitosan-conjugated mesoporous silica nanocomplex | *BIOMATERIALS* | Zhao RR | 47 |
| 7 | Cancer nanomedicine: progress, challenges and opportunities | *NATURE REVIEWS CANCER* | Shi JJ | 45 |
| 8 | Hepatocellular carcinoma | *NATURE REVIEWS DISEASE PRIMERS* | Llovet JM | 45 |
| 9 | Lenvatinib versus sorafenib in first-line treatment of patients with unresectable hepatocellular carcinoma: a randomised phase 3 non-inferiority trial | *LANCET* | Kudo M | 44 |
| 10 | Challenges in liver cancer and possible treatment approaches | *BIOCHIMICA ET BIOPHYSICA ACTA-REVIEWS ON CANCER* | Anwanwan D | 37 |
