# Supplementary material for: Seeing beyond words: nanotechnology in hepatocellular carcinoma - a bibliometric study
Source: Front Oncol. 2025 Jan 15;14:1487198. doi: 10.3389/fonc.2024.1487198 (PMC11774701; doi:10.3389/fonc.2024.1487198)
Supplement: Supplementary file 7 [file Table7.docx]

Table S7: Top 20 keywords associated with nanotechnology applications for Hepatocellular Carcinoma diagnosis and treatment.

| Rank | Keyword | Counts | Rank | Keyword | Counts |
| --- | --- | --- | --- | --- | --- |
| 1 | drug-delivery | 465 | 11 | chemotherapy | 193 |
| 2 | delivery | 428 | 12 | oxidative stress | 178 |
| 3 | apoptosis | 362 | 13 | drug delivery | 155 |
| 4 | therapy | 331 | 14 | co-delivery | 150 |
| 5 | cells | 330 | 15 | gold nanoparticles | 148 |
| 6 | doxorubicin | 301 | 16 | toxicity | 145 |
| 7 | expression | 246 | 17 | breast-cancer | 144 |
| 8 | cytotoxicity | 213 | 18 | release | 138 |
| 9 | in-vivo | 205 | 19 | nanomedicine | 129 |
| 10 | sorafenib | 196 | 20 | paclitaxel | 127 |
